# Tolvaptan for water retention in heart failure: a systematic review

**DOI:** 10.1186/s13643-023-02293-3

**Published:** 2023-07-29

**Authors:** Yujing Pan, Haoyang Li, Jin Gao, Zishuo Mi, Hao Chen, Ying Li

**Affiliations:** 1 Jiangsu Province Hospital of Chinese Medicine, Affiliated Hospital of Nanjing University of Chinese Medicine, Nanjing, 210023 China; 2 First Clinical Medical College, Nanjing University of Chinese Medicine, Nanjing, 210023 China; 3 College of Acupuncture and Chinese Tuina, Nanjing University of Chinese Medicine, Nanjing, 210023 China; 4 School of Nursing, Nanjing University of Chinese Medicine, Nanjing, 210023 China

**Keywords:** Tolvaptan, Heart failure, Water retention, Overview, GRADE, AMSTAR-2

## Abstract

**Objective:**

The purpose of this systematic review is to collect, appraise, and synthesize existing evidence from systematic reviews and meta-analyses (SRs/MAs) on the effectiveness of tolvaptan for water retention in heart failure.

**Methods:**

A comprehensive literature search was performed on PubMed, EMBASE, web of science, Cochrane reviews for SRs/Mas published between the databases’ establishment to November 17, 2021. All the records were managed with Endnote 20. Standardized forms were used to extract data. Revman 5.3 was used to make forest plots to show the characteristics of outcomes. The methodological and evidence quality were respectively evaluated by AMSTAR-2 (A MeaSurement Tool to Assess systematic Reviews 2) and GRADE (Grading of Recommendation of Assessment, Development, and Evaluation) system.

**Results:**

A total of 9 SRs/Mas between 2015 to 2020 met inclusion criteria. Serum sodium concentration and urine output were considered as primary outcomes and body weight change and all-cause mortality as second outcomes. Through conducting forest plots, it appeared that tolvaptan brought more positive effect than conventional therapies. It was pessimistic when it comes to the quality of the 9 studies. all the 9 articles were rated as low-quality because AMSTAR 2 evaluation showed that they each had at least one critical item (items 2, 4, 7, 9, 11, 13 and 15) defect. Besides, every article had a few non-critical item defects too. The result of GRADE assessment was not optimistic, so the overall quality of the evidences was low as well.

**Conclusion:**

Tolvaptan can be recommended for water retention in HF patients, but more evidence is needed.

**Supplementary Information:**

The online version contains supplementary material available at 10.1186/s13643-023-02293-3.

## Background

Heart failure (HF) is a complex clinical syndrome derived from any structural or functional impairment of ventricular filling or ejection of blood [1]. Currently, the absolute numbers of HF prevalent cases and years lived with disability(YLDs) have increased by 91.9% and 106.0% from 1990, respectively [2]. Sustained high volume loading due to water retention caused by reduced ejection is one of the most dangerous pathophysiological processes in HF, resulting in patients' dyspnea and even death. As the vital role of reliving congestion played, diuretics are the cornerstone of therapy in HF. According to current guidelines, for patients with HF who have fluid retention, diuretics are recommended to relieve congestion, improve symptoms and prevent worsening HF [1, 3], despite diuretic resistance and the potential adverse effects that would be related to electrolyte disturbance, impairment of renal function and activation of the renin angiotensin aldosterone system(RAAS) [4, 5].

Tolvaptan is a selective antagonist of the vasopressin V2 receptor whose action on the renal collecting ducts inhibits vasopressin-mediated water re-absorption [6]. Several clinical trials have provided mechanistic support for the symptomatic improvements and normalized serum sodium noted with tolvaptan in patients with decompensated HF [7–9]. Accordingly, tolvaptan may be appropriate for HF patients with electrolyte disturbance and poor response to conventional diuretics.

Currently, growing interest in the effects of tolvaptan on patients with water retention caused by HF has led to a continuous increase in the number of related SRs/MAs on this topic. Differences in the scopes, methods of analysis, and methodological quality of SRs/MAs can cause great confusion and make it difficult for policy makers and clinicians to access and interpret the available evidence and for researchers to know where knowledge gaps in the extant literature exist. In order to provide more reliable evidence, this study selected relevant SRs/MAs, concluded characteristics and conducted methodological and evidence quality assessment to form a comprehensive overview.

## Methods

The study was rigorously carried out according to the guidelines for systematic reviews provided by Cochrane Handbook and reported in compliance with the Preferred Reporting Items for Systematic Reviews and Meta-analysis (PRISMA) statement [10, 11].

### Inclusion and exclusion criteria

#### Type of studies

We included all peer-reviewed, full-reported SRs / MAs published in English of randomized controlled trials (RCTs) performed in humans that assessed the effectiveness of tolvaptan on water retention patients with HF. All duplicated documents were excluded.

#### Type of participants

Participants with HF should be diagnosed in accordance with 2021 ESC Guidelines for the diagnosis and treatment of acute and chronic heart failure [3]. Nationality, age, gender, and disease duration were not limited.

#### Type of interventions

The Interventions were tolvaptan or tolvaptan add-on therapy. The control groups should be placebo or other active controls like conventional diuretic therapy.

#### Type of outcomes

Primary outcomes were serum sodium concentration and urine output. Second outcomes contained weight change and all-cause mortality.

### Search strategy

PubMed, EMBASE, the Cochrane Database of Systematic Review and Web of Science Systematical were searched from inception up to November 17th, 2021. The detailed search strategy is in the Additional file 1.

### Data collection and extraction

Based on the inclusion and exclusion criteria, two authors (first two authors, Yujing Pan and Haoyang Li) independently screened all potential reviews for inclusion. A pre-designed sheet was used to extract relevant information including year, author, country, number of enrolled RCTs, participants, quality assessment tools, interventions and outcome measures.

### Methodological quality assessment

Two reviewers (first two authors, Yujing Pan and Haoyang Li) independently evaluated the methodological quality of included SRs/MAs with AMSTAR 2 from 16 domains [12]. Every item was rated in three levels, yes, partial yes or no. Based on the results of the critical appraisal, the enrolled reviews were rated as “high”, “moderate”, “low” or “critically low” (“High”, no or one non-critical weakness; “Moderate”, more than one non-critical weakness; “Low”, one critical flaw with or without non-critical weaknesses and “Critically low”, more than one critical flaw with or without non-critical weaknesses). All disagreements were resolved by consulting an experienced third reviewer (Hao Chen).

### Evidence quality assessment of the primary outcomes

The Grades of Recommendations, Assessment, Development, and Evaluation (GRADE) was employed to assess the evidence quality of the primary outcomes in the included SRs/MAs. The quality was classified as four levels—high, moderate, low, and very low [13]. GRADE identifies five key elements that influence quality of evidence and can be used for rating down one’s confidence in the estimates of intervention effects, which includes risk of bias, inconsistency, indirectness, imprecision and publication bias [14]. All the process of assessment was conducted by two reviewers (first two authors, Yujing Pan and Haoyang Li) independently and disagreements were resolved by a third experienced reviewer (Hao Chen).

### Statistical method

To avoid the potential heterogeneity of the included reviews, a qualitative analysis was carried out instead of a pooled result. All the relevant data was just presented in the forest plots without pooling analysis. Revman 5.3 was applied for formulating the forest plots.

### Patient and public involvement

No patient involved.

## Results

### Literature searching and study selection

**Fig. 1:**
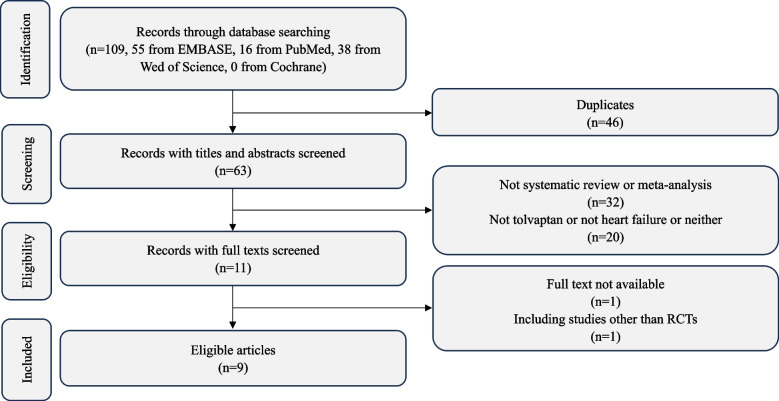
Flow chat of literature screening

The process of literature searching and screening is presented in Fig. 1. One hundred and nine references were screened for eligibility, of which 46 were removed for duplication. After reading the titles and abstracts, 32 more reviews were yielded for not being SRs/MAs and 20 others were excluded because they were not studies associated with the topic. The full texts of the other 11 references were identified in detail and 2 of them were excluded based on the inclusion criteria. Overall, 9 SRs/MAs met the inclusion criteria.

### Characteristics of the included reviews

General characteristics about year, author, country, number of enrolled RCTs, participants, interventions and outcome measures included in the 9 SRs/MAs along with the quality assessment tools are summarized in Table 1. The included reviews were published between 2015 and 2020 with 6 of them published after 2017. All eligible articles only enrolled RCTs. The number of RCTs in each review varied from 6 to 14 and the number of participants ranged from 669 to 13453. Seven reviews (7/9) used Cochrane risk of bias tool for the assessment of the risk of bias [15–21] and the rest used Jadad scale [22, 23]. The interventions in treatment groups were tolvaptan or tolvaptan add-on treatment while those were placebo or conventional treatment in control groups.

**Table 1:** Characteristics of the included studies

**Article IDs**	**Number of Included Studies**	**Number of Included participants**	**Treatment Group**	**Control Group**	**Tools for quality evaluation**	**Outcome measures**
Yang CJ 2015(CN) [23]	8	13453	Tolvaptan	placebo	Jadad scale	①③
Wang CB 2017(CN) [15]	6	746	Tolvaptan	placebo or active controls	Cochrane risk of bias tool	①③④
Wu MY 2019(CN) [16]	12	5793	Tolvaptan	Placebo	Cochrane risk of bias tool	①②③④
Ma G 2018(CN) [17]	7	937	Tolvaptan Add-on therapy	placebo or active controls	Cochrane risk of bias tool	①③④
Xiong B 2015(CN) [18]	10	5574	Tolvaptan	placebo or active controls	Cochrane risk of bias tool	①③④
Huang WI 2018(CN) [22]	6	669	Tolvaptan	placebo or active controls	Jadad scale	①②④
Kinugawa K 2018(JP) [19]	14	5991	Tolvaptan Add-on therapy	placebo or active controls	Cochrane risk of bias tool	①②③
Luo XD 2020(CN) [21]	12	5577	Tolvaptan Add-on therapy	placebo or active controls	Cochrane risk of bias tool	①②③④
Alskaf.E.E 2016(the UK) [20]	8	5385	Tolvaptan	Placebo	Cochrane risk of bias tool	①②③④

①Serum sodium concentration

②Urine output

③Weight loss

④All-cause mortality

*CN* China, *JP* Japan

### Methodological appraisal

**Fig. 2:**
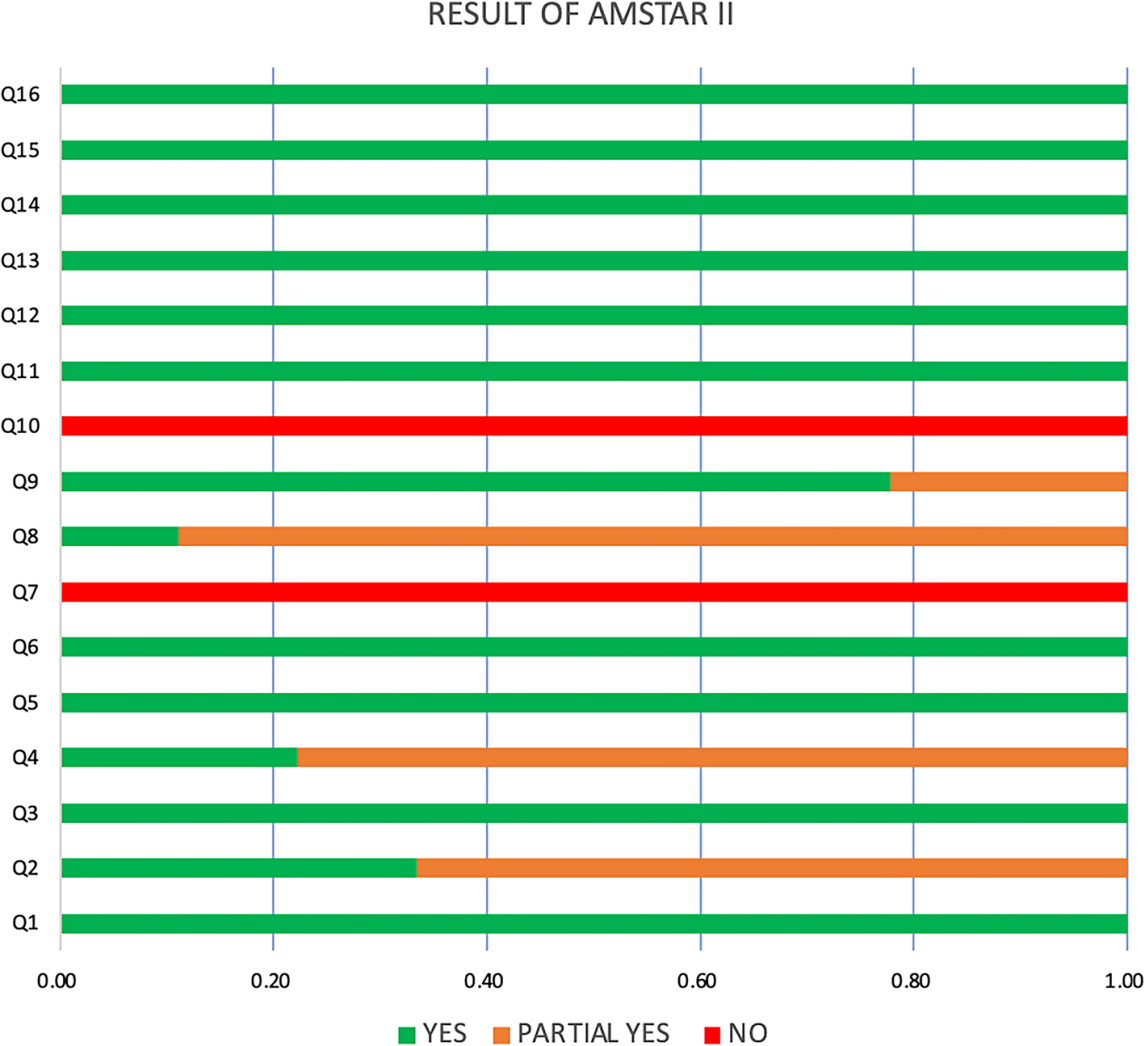
Summary of results of AMSTAR 2

A summary of the results of AMSTAR 2 has been offered in Fig. 2 formulated by EXCEL 2019. Overall, as none of the authors provided the lists of excluded studies and reasons for the exclusion, all studies were rated as “low”. That was a critical weakness (Domain 7). Besides, another weakness in all studies was no report on the sources of funding. Three of nine articles [16, 17, 20] were yes on Q2 and the remaining six articles [15, 18, 19, 21–23] were partial yes. On Q4 and Q8, the number of yes were respectively two [16, 21] and one [21].

### Quality of the evidence

Details were presented in Table 2. Because of the obvious risk of bias and serious imprecision detected in the SRs/Mas, the results of GRADE assessment showed that most of the SRs/MAs provided low or very low-quality evidence for tolvaptan on serum sodium concentration and urine output for patients with HF.

**Table 2:** Quality of evidence for primary outcomes

Reviews	Certainty assessment									*P*-value	Quality
	Outcomes	№ of participants		№ of studies	Risk of bias	Inconsistency	Indirectness	Imprecision	Other considerations		
		Experimental	Control								
Yang CJ 2015(CN) [23]	①	3535	3572	6	serious^a^	serious^b^	not serious	not serious	none	*P* < 0.0001	L
Wang CB 2017(CN) [15]	①	NA	NA	4	serious^a^	not serious	not serious	serious^c^	none	*P* = 0.711	L
Wu MY 2019(CN) [16]	①	2485	2148	8	not serious	not serious	not serious	not serious	none	*P* < 0.00001	H
	②	638	359	8	not serious	not serious	not serious	not serious	none	*P* < 0.00001	H
Ma G 2018(CN) [17]	①	51	52	2	serious^a^	serious^b^	not serious	very serious^c^	publication bias strongly suspected^d^	*P* = 0.04	VL
Xiong B 2015(CN) [18]	①	NA	NA	8	not serious	not serious	not serious	not serious	none	*P* = 0.000	H
Huang WI 2018(CN) [22]	①	264	298	5	not serious	not serious	not serious	serious^c^	none	*P* = 0.04	M
	②	242	254	4	not serious	not serious	not serious	serious^c^	none	*P* < 0.00001	M
Kinugawa K 2018(JP) [19]	①	1633	1615	6	serious^a^	serious^b^	not serious	not serious	none	*P* = 0.32	L
	②	226	228	5	not serious	not serious	not serious	serious^c^	none	*P* < 0.00001	M
Luo XD 2020(CN) [21]	①	2162	2188	6	not serious	serious^b^	not serious	not serious	none	*P* < 0.00001	M
	②	281	282	5	serious^a^	not serious	not serious	serious^c^	none	*P* < 0.00001	L
Alskaf.E.E 2016(the UK) [20]	①	345	346	5	serious^a^	not serious	not serious	serious^c^	none	*P* = 0.0001	L
	②	94	87	3	serious^a^	not serious	not serious	very serious^c^	publication bias strongly suspected^d^	*P* = 0.004	VL

①Serum sodium concentration

②Urine output

*H* High, *M* Moderate, *L* Low, *Vl* Very low

^a^The experimental design had a large bias in random, distributive findings, blinding or incomplete outcome date

^b^The confidence interval overlaps less, the heterogeneity test P, and the I^2^ was larger

^c^The confidence interval was not narrow enough, or the sample size was too small

^d^Funnel graph asymmetry, or fewer studies were included and there may have been greater publication bias

### Qualitative analysis of the effects

**Fig. 3:**
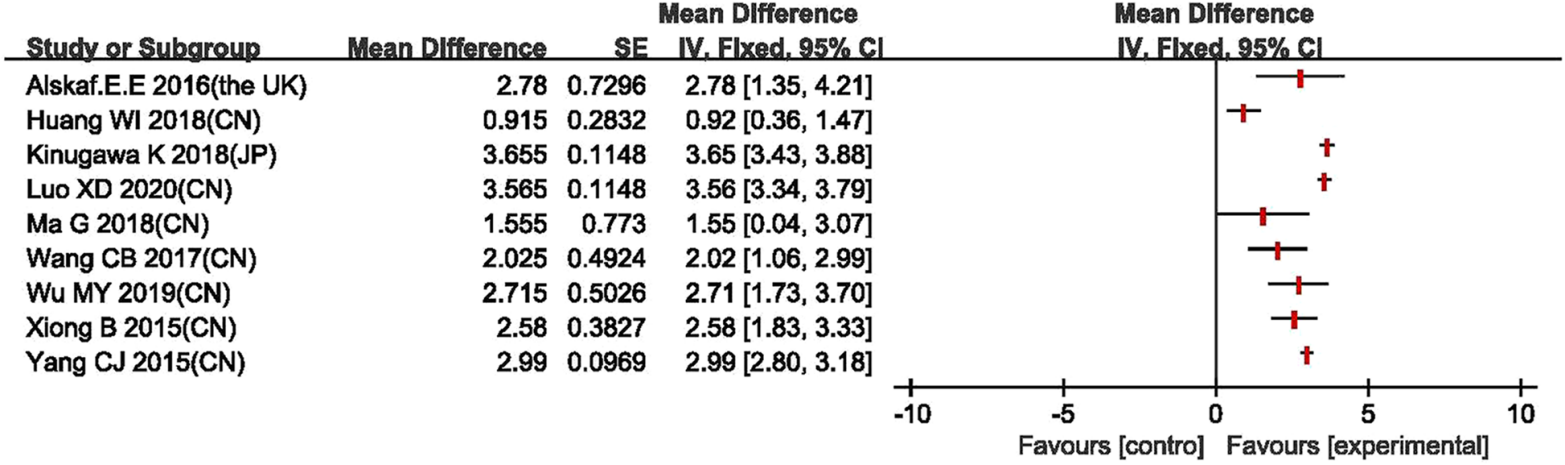
Forest plot of changes in serum sodium concentration

**Fig. 4:**
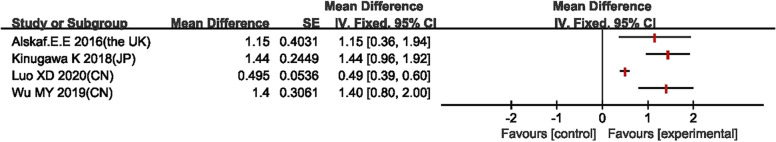
Forest plot of changes in urine output

**Fig. 5:**
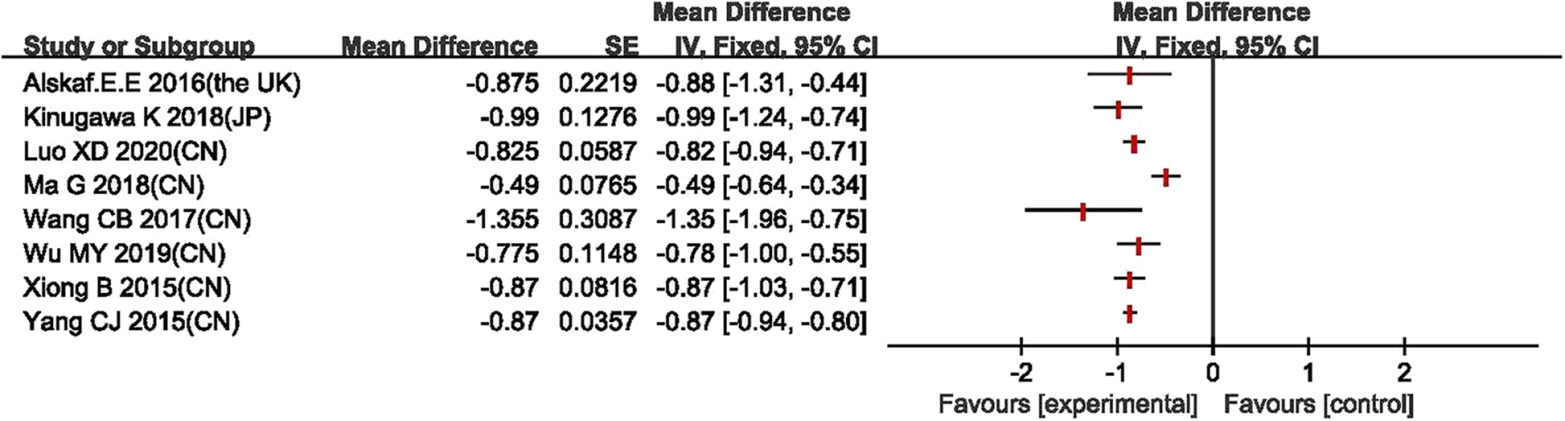
Forest plot of changes in weight loss

**Fig. 6:**
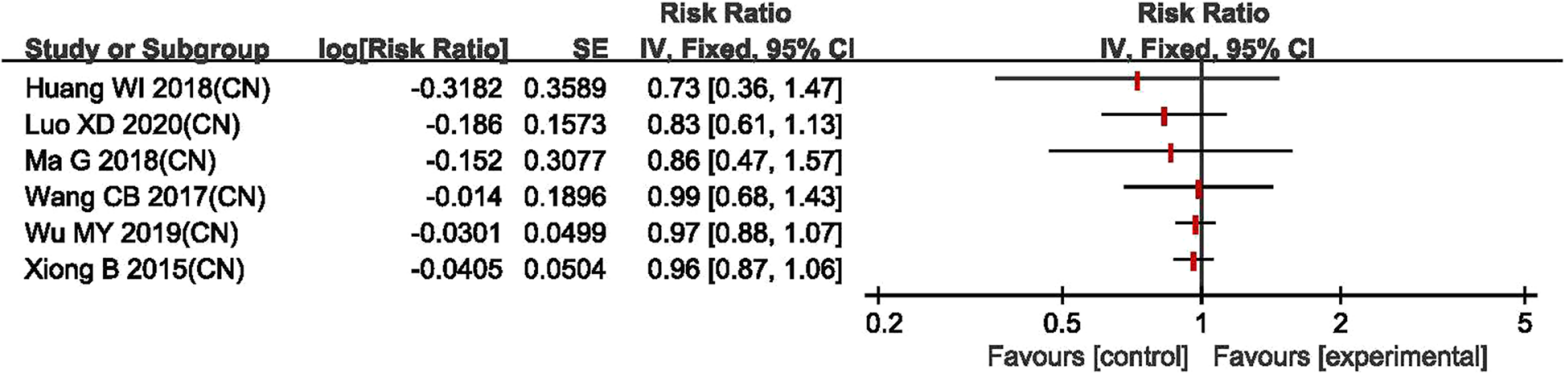
Forest plot of changes in all-cause mortality

The forest plots are in the Figs. 3, 4, 5, 6 for the four outcomes. Most studies reported that tolvaptan have advantage in modifying the level of serum sodium concentration, increasing urine output, lowering body weight and showing marginal effect in reducing rate of all-cause mortality. Overall, it is suggested that the effectiveness of tolvaptan was superior to placebo or other active controls (conventional diuretics).

### Primary outcomes

All nine studies reported serum sodium concentration as outcome. The forest plot showed that tolvaptan group was better than control group at increasing serum sodium concentration (Fig. 3). Similarly, five studies [16, 19–22] reported urine output; the forest plot shown that tolvaptan group was better than the control group in increasing urine output (Fig. 4).

### Secondary outcomes

For the secondary outcomes, eight of nine studies [15–21, 23] reported weight loss; the forest plot shown that reduction in body weight of tolvaptan group was significantly greater than those of the control group (Fig. 5). Seven studies [15–18, 20–22] reported all-cause mortality and the forest plot shown that the rate of all-cause mortality in tolvaptan group was not significantly lower than that of the control group (Fig. 6).

### Adverse effects

Eight of the nine reviews [15–18, 20–23] mentioned adverse events. Five of them [15, 17, 18, 20, 23] reported that tolvaptan showed no superiority on serious adverse events (worsening renal failure, worsening heart failure, length of hospitalization, cardiac events) to control group, but it did show no inferiority. Differently, one study [22] stated that tolvaptan could significantly lower the incidence of worsening renal failure. In particular, another study [21] draw the conclusion that low-dose tolvaptan add-on therapy could significantly reduce the incidence of worsening renal failure, whereas the opposite was shown in the high-dose group. Two [16, 23] emphasized the risk of thirst and it was reported that no significant differences were found between different dosages of tolvaptan [16].

## Discussion

### Main findings of this overview

The main findings indicate that tolvaptan could modify the level of serum sodium concentration, increase urine output and reduce body weight without affecting the rate of all-cause mortality. When it comes to side effects or tolvaptan-relevant adverse events, tolvaptan seemed relatively safe compared to conventional diuretics like furosemide, hydrochlorothiazide or mannitol, who can give rise to severe electrolyte disorder.

### Use of tolvaptan in heart failure

In 2021 ESC Guidelines for the diagnosis and treatment of acute and chronic heart failure, it is clearly stated that treatment of dilutional hyponatremia arising in heart failure should be based on water control with vasopressin antagonists; Tolvaptan, an orally active selective arginine vasopressin V2 receptor antagonist, is considered to increase serum sodium and diuresis in patients with persistent hyponatremia and congestion [3]. To date, the clinical application of tolvaptan mainly focus on improving the congestive symptoms, of which the most essential is water retention. Most patients hospitalized for decompensated HF are highly symptomatic due, in part, to volume overload, making decongestion through diuresis a primary treatment goal [24]. According to the work done by Teruhiko Imamura and his colleagues [25], in short-term use, the acute effect of tolvaptan contains an increasing urine volume and ameliorated dyspnea at day 1, followed by an increase in serum sodium concentration and a reduction in body weight, and in long-term use, patients with renal dysfunction or hyponatremia, or those with a history of repeated hospitalizations due to worsening HF are good candidates for tolvaptan therapy.

### The quality of current evidence

Although there were many optimistic reports and conclusions about tolvaptan as above, the methodological quality and evidence quality of included articles were generally suboptimal. The results of AMSTAR 2 evaluation suggested that more examinations should be done to determinate the reliability of these reports and conclusion. Given that the methodological quality of relevant SRs/MAs remained negative, it was very likely that the conclusion of these studies had a major departure from the real situation. GRADE assessment proved that generally, the evidence quality was not ideal, with 5 graded high, 11 moderate, 8 low and 5 very low. Taking the sample sizes into account, the evidence quality in six studies [16, 18–21, 23] was critical to judge the overall quality. Fortunately, they offered a relatively dependable conclusion, which saved the confidence of the whole evidence body from being very low. However, due to the limitations in the original trials, most outcomes were graded moderate, low quality and even very low quality. The effect of high-quality evidence is inadequate. There is much room for addressing the bias in random, distributive hiding, or blind during the RCT process; well-designed and implemented RCTs are considered gold standards for evaluating interventions to minimize or avoid bias [26].

### Tolvaptan alone could not lower the all-cause mortality

According to our findings, tolvaptan could effectively treat the serum sodium level while it could not bring down the all-cause mortality. Hyponatremia, is common and increasingly recognized as an independent prognostic marker that adversely affects mortality in heart failure. Persistent hyponatremia may indicate inadequate decongestion, which is related to poor outcomes in HF, especially in advanced HF [27–29]. It is a major cause of recurrent hospitalizations in patients with chronic heart failure and predicts death but is difficult to diagnose unequivocally [30]. Many studies demonstrated the strong prognostic value of hyponatremia being associated with a prolonged hospital length of stay, higher risk of readmission, and in hospital and after discharge mortality [31–35]. However, there is no clear indication yet whether hyponatremia in itself influences the prognosis or whether it just occurs more often in patients with heart failure [36]. Also, concrete evidence is still in absence to demonstrate the advantage of tolvaptan [33]. Combine the above, it suggested that hyponatremia may not play a causal role in mortality in patients with heart failure. That can explain why treating hyponatremia with tolvaptan in isolation could not lower the mortality significantly. Hence, to measure the therapeutic effect of tolvaptan on HF patients, rate of hospitalization length and recurrent hospitalization may be better choice in follow-up studies, which are more directly associated with hyponatremia [34, 35, 37]. From the perspective of methods, the treatment intervention in this study enrolled not only tolvaptan but also tolvaptan add-on therapy, which could bring about potential bias. In general, no firm conclusion can be draw from current evidence.

### Agreements and disagreements with other studies

To our knowledge, this study is the first overview on the efficacy of tolvaptan for water retention in HF patients. Our finding was in line with the six reviews of larger sample size [16, 18–21, 23]. However, tolvaptan-related adverse effects were unclear because the reports were inadequate.

### Potential bias and limitations in the progress of this overview

There are bias and limitations: 1) There might be articles ready to publish but missed, leading to possible publication bias; 2) Only studies in English were included and this may cause selection bias; 3) The participants of the articles were overwhelmingly Asians and it is likely that the efficacy of tolvaptan differs markedly due to objective differences in populations worldwide; 4) The included literatures were generally of low quality and could not provide a clear answer to the actual utility of tolvaptan; 5) For the sake of the methodological immaturity in overview, the included studies might employ repeated studies.

### Implications

Based on the analysis above, we believed that the effect of tolvaptan on water retention in heart failure temporarily cannot draw definitive and reliable conclusions. SRs/MAs of high quality are crucial to ensure validity, clarity and accurate comprehension of evidence. Therefore, it is hard to draw a clear conclusion of the efficacy of tolvaptan for HF patients with water retention in that based on the results of AMSTAR 2 and GRADE assessment, the methodology quality was low and the evidence quality was not very pleasing. Additionally, most SRs/MAs insisted that an increasing number of large sample size RCTS should be performed with high-quality methodology.

## Conclusion

Tolvaptan can be recommended for water retention in HF patients, but more evidence is needed. More rigorous RCTs adhering to international guideline are necessary to reach a definite conclusion based on compelling evidence.

## Supplementary Information

**Additional file 1. **Search strategy.

**Additional file 2: Supplementary Table 1.** Results of the AMSTAR-2 assessments.

## Data Availability

The original contributions presented in the study are included in the article/supplementary material, further inquiries can be directed to the corresponding authors.

## Abbreviations

SRs/MAs: Systematic reviews and meta-analyses
AMSTAR-2: A MeaSurement Tool to Assess systematic Reviews 2
GRADE: Grading of Recommendation of Assessment, Development, and Evaluation system
HF: Heart failure
YLDs: Years lived with disability
RCTs: Randomized controlled trials
RAAS: Renin angiotensin aldosterone system

## References

[1] Heidenreich PA, Bozkurt B, Aguilar D, Allen LA, Byun JJ, Colvin MM (2022). 2022 AHA/ACC/HFSA Guideline for the Management of Heart Failure: A Report of the American College of Cardiology/American Heart Association Joint Committee on Clinical Practice Guidelines. J Am Coll Cardiol.

[2] NL B, Id- Orcid X, W Z, J S, A AM, D L, et al. Burden of heart failure and underlying causes in 195 countries and territories. D - 101564430. Eur J Prev Cardiol. 2021;28(15):1682–90. 10.1093/eurjpc/zwaa147. Issue - 2047-4881 (Electronic) Pages T - ppublish. 10.1093/eurjpc/zwaa147 33571994

[3] McDonagh TA, Metra M, Adamo M, Gardner RS, Baumbach A, Böhm M (2021). 2021 ESC Guidelines for the diagnosis and treatment of acute and chronic heart failure. Eur Heart J.

[4] Felker GM, Ellison DH, Mullens W, Cox ZL, Testani JM (2020). Diuretic therapy for patients with heart failure: JACC state-of-the-art review. J Am Coll Cardiol.

[5] Mullens W, Damman K, Harjola VP, Mebazaa A, Brunner-La Rocca HP, Martens P (2019). The use of diuretics in heart failure with congestion - a position statement from the Heart Failure Association of the European Society of Cardiology. Eur J Heart Fail.

[6] Veeraveedu PT, Watanabe K, Ma M, Palaniyandi SS, Yamaguchi K, Kodama M (2008). Effects of V2-receptor antagonist tolvaptan and the loop diuretic furosemide in rats with heart failure. Biochem Pharmacol.

[7] Gheorghiade M, Niazi I, Ouyang J, Czerwiec F, Kambayashi J, Zampino M (2003). Vasopressin V2-receptor blockade with tolvaptan in patients with chronic heart failure: results from a double-blind, randomized trial. Circulation.

[8] Konstam MA, Gheorghiade M, Burnett JC, Grinfeld L, Maggioni AP, Swedberg K (2007). Effects of oral tolvaptan in patients hospitalized for worsening heart failure: the EVEREST Outcome Trial. JAMA.

[9] Udelson JE, Orlandi C, Ouyang J, Krasa H, Zimmer CA, Frivold G (2008). Acute hemodynamic effects of tolvaptan, a vasopressin V2 receptor blocker, in patients with symptomatic heart failure and systolic dysfunction: an international, multicenter, randomized, placebo-controlled trial. J Am Coll Cardiol.

[10] Higgins JPT, Thomas J, Chandler J, Cumpston M, Li T, Page MJ, et al. Cochrane Handbook for Systematic Reviews of Interventions version 6.3 (updated February 2022). Cochrane; 2022. Available from: www.training.cochrane.org/handbook.

[11] Liberati A, Altman DG, Tetzlaff J, Mulrow C, Gøtzsche PC, Ioannidis JP (2009). The PRISMA statement for reporting systematic reviews and meta-analyses of studies that evaluate healthcare interventions: explanation and elaboration. BMJ.

[12] Shea BJ, Reeves BC, Wells G, Thuku M, Hamel C, Moran J (2017). AMSTAR 2: a critical appraisal tool for systematic reviews that include randomised or non-randomised studies of healthcare interventions, or both. BMJ.

[13] Guyatt GH, Oxman AD, Vist GE, Kunz R, Falck-Ytter Y, Alonso-Coello P (2008). GRADE: an emerging consensus on rating quality of evidence and strength of recommendations. BMJ.

[14] Balshem H, Helfand M, Schünemann HJ, Oxman AD, Kunz R, Brozek J (2011). GRADE guidelines: 3. Rating the quality of evidence. J Clin Epidemiol.

[15] Wang CB, Xiong B, Cai L (2017). Effects of Tolvaptan in patients with acute heart failure: a systematic review and meta-analysis. BMC Cardiovasc Disord..

[16] Wu MY, Chen TT, Chen YC, Tarng DC, Wu YC, Lin HH (2019). Effects and safety of oral tolvaptan in patients with congestive heart failure: a systematic review and network meta-analysis (vol 12, e0184380, 2017). PLoS ONE.

[17] Ma G, Ma XX, Wang GL, Teng W, Hui XZ (2019). Effects of tolvaptan add-on therapy in patients with acute heart failure: meta-analysis on randomised controlled trials. BMJ Open.

[18] Xiong B, Huang YW, Tan J, Yao YQ, Wang CB, Qian J (2015). The short-term and long-term effects of tolvaptan in patients with heart failure: a meta-analysis of randomized controlled trials. Heart Fail Rev.

[19] Kinugawa K, Sato N, Inomata T (2018). Effects of Tolvaptan on volume overload in patients with heart failure. Int Heart J.

[20] Alskaf EE, Tridente AA, Al-Mohammad AA (2016). Tolvaptan for heart failure, systematic review and meta-analysis of trials. J Cardiovasc Pharmacol.

[21] Luo XD, Jin Q, Wu YQ (2020). Tolvaptan add-on therapy in patients with acute heart failure: a systematic review and meta-analysis. Pharmacol Res Perspect.

[22] Huang WI, Yang Y, Yang J, Yang J, Wang HB, Xiong XI (2018). Use of tolvaptan vs. furosemide in older patients with heart failure Meta-analysis of randomized controlled trials. Herz.

[23] Yang CJ, Yang J, Yang J, Fan ZX (2015). Arginine vasopressin antagonist tolvaptan in the treatment of heart failure: a meta-analysis of randomized controlled trials. Int J Clin Exp Med.

[24] Gunderson EG, Lillyblad MP, Fine M, Vardeny O, Berei TJ (2019). Tolvaptan for volume management in heart failure. Pharmacotherapy.

[25] Imamura T, Kinugawa K (2019). Update of acute and long-term tolvaptan therapy. J Cardiol.

[26] Schulz KF, Altman DG, Moher D (2010). CONSORT 2010 statement: updated guidelines for reporting parallel group randomized trials. Ann Intern Med.

[27] Jao GT, Chiong JR. Hyponatremia in acute decompensated heart failure: mechanisms, prognosis, and treatment options. Clinical cardiology. 2010;33(11):666–71. 10.1002/clc.20822 PMC6653540 21089110

[28] Palazzuoli A, Testani JM, Ruocco G, Pellegrini M, Ronco C, Nuti R (2016). Different diuretic dose and response in acute decompensated heart failure: clinical characteristics and prognostic significance. Int J Cardiol.

[29] Testani JM, Chen J, McCauley BD, Kimmel SE, Shannon RP (2010). Potential effects of aggressive decongestion during the treatment of decompensated heart failure on renal function and survival. Circulation.

[30] Wilcox CS, Testani JM, Pitt B (2020). Pathophysiology of diuretic resistance and its implications for the management of chronic heart failure. Hypertension (Dallas, Tex : 1979).

[31] Gheorghiade M, Rossi JS, Cotts W, Shin DD, Hellkamp AS, Piña IL (2007). Characterization and prognostic value of persistent hyponatremia in patients with severe heart failure in the ESCAPE Trial. Arch Intern Med.

[32] Omar HR, Charnigo R, Guglin M (2017). Prognostic significance of discharge hyponatremia in heart failure patients with normal admission sodium (from the ESCAPE Trial). Am J Cardiol.

[33] Wang J, Zhou W, Yin X (2019). Improvement of hyponatremia is associated with lower mortality risk in patients with acute decompensated heart failure: a meta-analysis of cohort studies. Heart Fail Rev.

[34] Patel YR, Kurgansky KE, Imran TF, Orkaby AR, McLean RR, Ho YL (2018). Prognostic significance of baseline serum sodium in heart failure with preserved ejection fraction. J Am Heart Assoc.

[35] Su Y, Ma M, Zhang H, Pan X, Zhang X, Zhang F (2020). Prognostic value of serum hyponatremia for outcomes in patients with heart failure with preserved ejection fraction:an observational cohort study. Exp Ther Med.

[36] Şorodoc V, Asaftei A, Puha G, Ceasovschih A, Lionte C, Sîrbu O (2023). Management of hyponatremia in heart failure: practical considerations. J Pers Med.

[37] Mitani H, Funakubo M, Sato N, Murayama H, Rached RA, Matsui N (2020). In-hospital resource utilization, worsening heart failure, and factors associated with length of hospital stay in patients with hospitalized heart failure: a Japanese database cohort study. J Cardiol.

