# Supplementary material for: Tolvaptan for water retention in heart failure: a systematic review
Source: Syst Rev. 2023 Jul 29;12:130. doi: 10.1186/s13643-023-02293-3 (PMC10386224; doi:10.1186/s13643-023-02293-3)
Supplement: Supplementary file 1 — Additional file 1. Search strategy. [file 13643_2023_2293_MOESM1_ESM.pdf]

Search Strategy (PubMed as example) :

#1 Search: Tolvaptan[Title/Abstract]

#2 Search: "Tolvaptan"[Mesh] Sort by: Most Recent

#3 Search: "Heart Failure"[Mesh] Sort by: Most Recent

#4 Search: "heart failure"[Title/Abstract]

#5 Search: "cardio-renal syndrome"[Title/Abstract]

#6 Search: "heart failure, diastolic"[Title/Abstract]

#7 Search: "heart failure, systolic"[Title/Abstract]

#8 Search: "edema, cardiac"[Title/Abstract]

#9 Search: "dyspnea, paroxysmal"[Title/Abstract]

#10 Search: #1 OR #2

#11 Search: #3 OR #4 OR #5 OR #6 OR #7 OR #8 OR #9

#12 Search: #11 AND #10

#13 Search: #11 AND #10 Filters: Systematic Review

#14 Search: #11 AND #10 Filters: Meta-Analysis, Systematic Review
